# Pattern of Internet Use by Iranian Nursing Students. Facilitators and Barriers

**DOI:** 10.17533/udea.iee.v37n2e06

**Published:** 2019-09-19

**Authors:** Fatemeh Shirazi, Shiva Heidari, Sorur Javanmardi Fard, Fariba Ghodsbin

**Affiliations:** 1 Nurse, Ph.D. Assistant professor, Shiraz University of Medical Sciences, Shiraz, Iran. Email: shirazi_1393@yahoo.com Shiraz University of Medical Sciences Shiraz Iran shirazi_1393@yahoo.com; 2 Nurse, M.Sc. Instructor professor, Urmia branch, Islamic Azad University, Urmia, Iran. Email:communityhn@gmail.com. Corresponding author. Islamic Azad University Islamic Azad University Urmia Iran communityhn@gmail.com; 3 Nurse, M.Sc. Instructor professor, School of Nursing and Midwifery, Shiraz University of Medical Sciences, Shiraz, Iran. Email: soror.javanmardi@yahoo.com Shiraz University of Medical Sciences Shiraz Iran soror.javanmardi@yahoo.com; 4 Nurse, M.Sc. Assistant Professor, Quality Improvement in Clinical Education Research Center, School of Nursing and Midwifery, Shiraz University of Medical Sciences, Shiraz, Iran. Email: ghodsbin@sums.ac.ir Shiraz University of Medical Sciences Shiraz Iran ghodsbin@sums.ac.ir

**Keywords:** computers, cross-sectional studies, information technology, information storage and retrieval, Internet, students, nursing., computadores, estudios transversales, tecnología de la información, almacenamiento y recuperación de la información, Internet, estudiantes de enfermería., computadores, estudos transversais, tecnologia da informação, armazenamento e recuperação da informação, Internet, estudantes de enfermagem.

## Abstract

**Objective.:**

To evaluate the pattern of internet use and factors that facilitate or dissuade its use among nursing students from a university in Urmia, Iran.

**Methods.:**

A cross-sectional, descriptive study was conducted with 162 nursing students selected through simple random sampling.

**Results.:**

The findings indicated that 49.1% of the students used the internet from 15 to 60 min per day. The principal use of the internet was to search for scientific content in the Web. Factors that facilitated internet use were "ease of use" and "Access to experts to solve problems and answer questions", while the dissuasive factors were "lack of concentration", "cost of internet services", and preference for information provided by professors or available directly in textbooks. Internet use by the students was related with the use of this tool in classroom activities and with English fluency.

**Conclusion.:**

Students have an internet use pattern aimed at self-study that should be strengthened with knowledge of English, assignments online, familiarization with the use of electronic databases, and other strategies to motivate them to use this technology with greater frequency.

## Resumen

**Objetivo.:**

Evaluar el patrón de uso del Internet y los factores que facilitan o disuaden su uso entre los estudiantes de enfermería de una universidad en Urmia, Irán.

**Métodos.:**

Se realizó un estudio descriptivo de corte transversal en 162 estudiantes de enfermería seleccionados a través de un muestreo aleatorio simple.

**Resultados.:**

Los hallazgos indicaron que el 49,1 % de los estudiantes usaron Internet de 15 a 60 minutos por día. El principal uso del Internet fue la búsqueda de contenido científico en la Web. Los factores que facilitaron el uso de internet fueron la "facilidad de uso" y el "acceso a expertos para resolver problemas y responder preguntas", mientras que los factores disuasivos fueron la "falta de concentración", el "costo de los servicios de Internet" y la preferencia por la información suministrada por profesores o disponible directamente en los libros. El uso del Internet por los estudiantes estuvo relacionado con empleo de esta herramienta en las actividades del salón de clase y con el dominio del idioma inglés.

**Conclusión.:**

Los estudiantes tienen un patrón de uso del Internet encaminado al auto-estudio que debe fortalecerse en conocimiento del idioma inglés, asignaturas en línea, familiarización con el uso de bases electrónicas y otras estrategias para motivarlos a usar esta tecnología con mayor frecuencia.

## Resumo

**Objetivo.:**

Avaliar o padrão de uso da Internet e os fatores que facilitam ou dissuadem seu uso entre os estudantes de enfermagem de uma universidade em Urmia, Irã.

**Métodos.:**

Se realizou um estudo descritivo de corte transversal em 162 estudantes de enfermagem que foram selecionados através de uma amostra aleatória simples.

**Resultados.:**

As descobertas indicaram que 49.1% dos estudantes usaram Internet de 15 a 60 minutos por dia. O principal uso da Internet foi a busca de conteúdo científico na Web. Os fatores que facilitaram o uso de internet foram a "facilidade de uso" e o "acesso à especialistas para resolver problemas e responder perguntas", enquanto que os fatores dissuasivos foram a "falta de concentração", o "custo dos serviços de Internet" e a preferência pela informação subministrada por professores ou disponíveis diretamente nos livros. O uso da Internet pelos estudantes esteve relacionado com emprego desta ferramenta nas atividades da aula e com o domínio do idioma inglês.

**Conclusão.:**

Os estudantes têm um padrão de uso da Internet encaminhado ao auto-estudo que deve fortalecer-se em conhecimento do idioma inglês, matérias em linha, familiarização com o uso de bases eletrônicas, e outras estratégias para motivá-los a usar com maior frequência esta tecnologia.

## Introduction

Development of information technology and its use has had deep effects on different aspects of human life, including education. In recent decades, having access to information technology and the ability to use it, became an essential issue for those living in informational societies.(1) Educational systems globally have great needs to use information and communication technologies for education; meantime, the internet has gained a special position. Computers and internet, as educational technologies, can meet the information and research needs of students.(2) 

Having knowledge and skills to use the internet, helps people to use information effectively; conduct searches, and produce and assess information. Information resources on the internet, such as databases, websites, and weblogs provide a positive environment for searching and studying. Additionally, they act as facilitators for interpreting, integrating, and applying knowledge in different fields of learning.(3) Numerous advantages of using the internet in research, communication and education have motivated universities and higher education institutions to provide accessibility to this massive network for their students and staff. 

Some of the advantages of the internet network, like having access to information resources and scientific findings, sharing knowledge, scientific communication between scientists, distance learning, access to virtual libraries, etc., have made it more important than it was supposed. Furthermore, some limitations, such as financial limitations of universities, place and time limitations to access printed resources and libraries made the internet the easiest means of access to scientific information. Nowadays, access to traditional library catalogues and resources is possible through the internet.(4) Being familiar with computers and the internet is one of the essential competencies of nursing students that will be used to search for information through websites and software. Having computer and internet literacy is essential to gain learning goals.(5) Nursing students can use the internet and computer software for different goals, such as doing homework, preparing seminars, watching films, communicating with friends and instructors, searching for information to update knowledge and evidenced-based practices, and to use library databases.(6) These skills are also important for their future professional assignments. Therefore, proficiency in using internet technology (IT) is an essential need in nursing education.(5) Researchers believe that using IT must become a critical part of nursing education programs in the future.(7)

Acquiring internet skills is not only essential for nursing education and studies, but it is also important to help patients to look for suitable answers for their health questions. Nurses working in health care settings have a critical educational responsibility in guiding patients who increasingly access online resources of health information to make decisions.(8) Therefore, it is essential for nurses and nursing students to have access to online health databases and also be familiar with informational resources to help patients and their families capture proper and reliable information.(9)

In recent years, several studies have been conducted to evaluate students’ and instructors’ knowledge and skills about using computers and the internet in different fields. As recommended by the findings, people in developed countries, in particular, use the internet to search for solutions to problems in their studies, and professional and personal matters. The popularity of the internet and its growing use among adolescents and youth is undeniable. These groups use the internet as their main source of information.(10) Results by different studies have indicated that using IT is related to a variety of factors, like age, sex, English fluency, computer and internet skills, feeling ease and comfort in using IT, among others.(4) Taking into account the results of the studies mentioned on the one hand and the key role of education in modern society, on the other hand, it seems that using IT in academic centers is essential. Considering the weight of using computers, the internet, and databases in performing scientific and practical activities, it is vital to assess the pattern of using computers and the internet in universities and the challenges in this field. The results may be helpful to facilitate and promote using this technology. Thus, this study is aimed at assessing the pattern of using computers and the internet, and its facilitators, and deterrents in nursing students. 

## Methods

This is a descriptive cross-sectional study on 169 nursing students in Islamic Azad University of Urmia, Urmia, Iran. The participants were selected through a simple random sampling using a table of random numbers of all nursing students, from October to December 2016. The sample size was determined based on similar studies,(6,11) with type I error of 0.05 and test power of 0.8.

The data-gathering tool was a researcher-designed questionnaire with 36 statements designed in three sections. The first section collected demographic and educational information, like age, educational level, place of residence, marital status, English fluency, and cumulative grade point average (GPA), calculated by dividing the total amount of grade points earned by the total amount of credit hours. The second section gathered information about using computers, places of access, and computer skills (*e.g.*, typing, installing software, file management, and ability to understand software/hardware technical terms). Computer skills were assessed by using seven statements designed based on a Likert scale - excellent (5) to poor (1) - and obtainable score ranging from 7 to 35. The third part of the questionnaire collected data about using the internet, time spent on the net, place of access, extent of internet activities, facilitators and deterrents, and the mostly visited Iranian and non-Iranian websites.

The face validity and content validity of the questionnaire were confirmed by 10 experienced academic members (two assistant professors and eight instructors). The item-level content validity index (I-CVI) of almost all items was >0.8. Good reliability of the questionnaire was also ensured by using test re test (r=0.78).

The questionnaire was provided to the participants by the researcher and they were allowed to fill out the questionnaire anonymously. The participants were briefly introduced to the necessity and nature of the study, and were allowed to participate voluntarily. This study was approved by the Research Council and the Ethics Committee of the Urmia branch of Islamic Azad University, Urmia, Iran (Code: 35411).

The data gathered was analyzed in SPSS (22) using descriptive and analytical statistical tests, such as mean, standard deviation, and correlation test.

## Results

All the participants were female with mean age of 21±0.7. The majority of the participants (75.5%) were single and 49.7% had mediocre financial status. Average GPA of the participants was 15.90±9.2 and more than half of them (57.7%) reported not having good command of English.

The findings showed that about 51.5% of the students had passed computer courses and 52.1% of them had mentioned that they had adequate access to computers in their faculty. With regard to access to the internet, most of the participants (54.2%) used high-speed internet at the Faculty of Midwifery and Nursing. As far as access to computers and the internet in the dormitories, 22.1% had no access to computers and 30% had no access to the internet. The average time spent on the computer was 6.7±8.7 h per week and the participants’ file management skills (copying, saving, creating, deleting, and printing) was greater than their other computer operating skills; and their skills on security of computers against viruses, hackers, and others were lower than the other computer operating skills (Table 1). 

**Table 1:** Mean score of the participants’ computer and internet skills

Skills	M±SD
Computer skills	
File management (copying, saving, creating, deleting, and printing)	3.20± 1.25
Typing and using the keyboard	2.98± 1.20
Troubleshooting simple malfunctions	2.61± 1.11
Using accessories, such as printer, scanner, etc.	2.29± 1.25
Understanding hardware/software technical terms	2.25± 1.07
Installing new software	2.22± 1.30
Protecting computer against viruses, hackers’ attacks, and others	2.14± 1.15
Internet skills	
Working with search engines: Yahoo and Google	4.68± 1.52
Saving information from the internet in the computer	4.33± 1.50
Checking e-mails	4.11± 1.97
Searching for articles and books	4.11± 1.54
Sending e-mail	3.68± 1.92
Signing up	3.60± 1.99
Attaching files to e-mail	3.44± 1.93
Using chat rooms	3.30± 1.76
Using e-libraries	3.28± 1.99

In using the Internet, 49.1% of the participants mentioned that they tend to be online around 15 to 60 min every day. In addition, 70.6% of the participants reported using the internet before entering the university and a large group of the participants (83%) expressed their need for further internet training. For searching in the internet, 31.9% mentioned that they had learned it from their families, 19.5% learned it through self-learning, 15.3% participated in non-academic training courses, 14.7% learned it from their classmates; and only 9.2% participated in academic training courses. The majority of the participants (58.3%) stated that they were mostly online at home and 23.9% mentioned they tended to use the internet in the Faculty. As to the purpose for using the internet, searching for scientific material was at the top and using video-chat was at the bottom (Table 2).

**Table 2:** Average time spent on the internet for different purposes

Different tasks	M±SD
Searching for scientific material	3.45±1.20
Personal interests	3.42±1.09
Searching for general material	3.01±1.19
Doing research work	2.95±1.28
Checking e-mail	2.79±1.53
Searching for technical information	2.77±1.24
Entertainment	2.75±1.32
Checking news	2.24±1.18
Downloading software	2.32±1.36
Chatting	2.08±1.45
Marketing and searching for products	1.94±1.21
Checking seminars and scientific events	1.68±0.94
Video chat	1.58±1.05

Amongst the Iranian and non-Iranian websites, the Scientific Information Database (SID) (22%) and Google (86%) were the websites visited most. The main facilitating factors in using the internet were “ease and comfort in using the internet” and “Access to experts to solve probable problems and answer probable questions”, however, “lack of concentration in using the internet”, “internet connection problems” and “cost of internet services” were the main deterrents (Table 3). 

**Table 3:** Facilitators and barriers in using the internet

	M±SD
Facilitators	
Ease and comfort using computers and the Internet	2.50±1.13
Access to experts to solve probable problems and answer probable questions	2.37±1.32
Adding internet training to the curriculum	2.32±1.30
Introduction of useful websites by the professors and friends	2.26±1.12
Familiarity with computers	2.24±1.31
Holding internet training courses in the Faculty	2.24±1.29
Feeling the need to use the internet	2.22±1.13
Availability of a computer facility with internet connection in the faculty/hospital	2.16±1.16
Having adequate knowledge about how to do a search on the internet	2.16±1.15
Barriers	
Lack of concentration in using the internet	3.08±1.67
Internet connection problems (low connection speed, disconnections, and etc.)	2.39±1.23
Cost of internet services	2.60±1.33
Preference for information collected from professors and experts	2.03±1.60
Preference to collect information from books and journals	1.77±0.42
Necessity to reserve internet in the university	1.93±0.25
Lack of self-confidence in using the internet	1.93±0.20
Distrust in the online information	1.92±0.26
Not feeling the need to use the internet	1.90±0.30
Disconnection	1.87±0.33
Lack of sufficient access to the internet	1.82±0.37
Failure to find the needed materials on the internet	1.81±0.38
Limits and restrictions on time using the internet	1.78±0.41
Lack of sufficient skills and experience in using computers and the internet	1.77±0.42
Lack of adequate time for internet surfing	1.70±0.45

Computer and Internet skills were significantly related to command of English, time spent on the computer, time spent on the internet, and using the internet as part of the curriculum, but these had no association with the GPA (Table 4). 

**Table 4:** Correlation between the internet and computer skills and educational variables

	Internet	skills	Computer	skills
Educational Variables	p	r	p	r
				
Grade point average	0.76	0.03	0.48	0.06
English fluency	<0.001	0.48	<0.001	0.52
Time spent on the computer	<0.001	0.38	<0.001	0.26
Time spent on the internet	0.03	0.24	<0.001	0.26
Using the internet as part of classroom activities	<0.001	0.49	<0.001	0.56

## Discussion

In the present study, the IT use pattern, as well as its facilitators and deterrents in the Nursing and Midwifery Faculty students from the Islamic Azad University of Urmia were examined. Based on the findings, the participants stated that access to computers and the internet in the Faculty was adequate, while most of them did not have access to computers and the internet in the dormitories. Saeidi *et al*.,(11) conducted a study on nursing students and reported that 44.8% had access to computers and 47.6% access to the internet, which was an acceptable access level to these facilities. Similar to our results, Kumar *et al.* (12) reported an access level of 59.9% to computers among students, while this figure in the work by Abtahi *et al.,*(13) was 33%. Access to the internet in Jamshidi *et al*.,(14) and in Jadoon *et al.,*(15) was 63.5% and 83.97%, respectively. Saeidi *et al.,*(11) argued that these findings indicate high level of access to computers and the internet in societies and that level of access to online information was a necessity of modern life, especially for students. The findings of the present study showed that most of the participants used the Google search engine, which is in line with Saeidi *et al., (*11) Scott *et al*.,(16) and Maleki *et al.*(17) User friendly interface and variety of capabilities of the Google search engine makes it the first choice for most internet users to search for information on the internet. 

As to the purposes for using the internet, the participants mentioned searching for scientific material as the main purpose and video chat as the lowest priority in using the internet. Saeidi *et al*.,(11) reported that searching for scientific articles was the main activity of the subjects on the net and checking e-mail was the least important activity. In today's world, the internet has become one of the main tools to access information. Along with its rapid expansion in our personal lives, the internet has brought substantial change in academic and research activities.

Increased computer and internet usage has influenced medical education, too. The Internet has made medical knowledge accessible for everyone around the world.(18) Unnikrishnan *et al*.,(19) also reported that most students use the internet for searching, obtaining general information, and for fun. The study by Marrof *et al.,*(20) showed that the greatest use of the internet was for communicating with others and for fun; and only one fiftens of the participants used the internet to search for articles. Jadoon *et al.,*(15) showed that 61% of the paricipants used the internet for personal and academic activities and only 11% used the internet merely for academic activities.

Our results showed that the majority of the participants had learned internet surfing via their family, self-learning, attending nonacademic courses, classmates, and academic courses held at the university. Mashhadi *et al.,*(21) showed that friends and colleagues, self-learning, and self-study were the main ways of learning how to use the internet. Sajadi *et al.,(*22) noted that most of the participants had obtained internet skills outside the academic environment. Shirazi *et al*.,(23) also indicated that nursing students performed different activities, such as interaction with others to construct their knowledge through their self-directed learning. Although it is possible to learn how to use computers and the internet through self-study and self-learning, the professional use of these tools certainly needs education. It is necessery to schedule regular classes under the suprevision of experienced instructors at schools.

The results showed that computer and internet skills were significantly related to the users’ knowledge of English, time spent on the computer and the internet, and using the internet as part of the curriculum. However, there were no significant relationships between age and educational performance and other variables. Asadi *et al*.,(24) reported that the number of areas using IT, as required by professors, was significantly related to the knowledge and skills of using computers, Internet, and command of English. Saeedi *et al*.,(11) showed that internet and computer skills were significantly related to the time spent on computers and the internet. Mashhadi *et al*.,(21) found in their study that age, computer and Internet using skills, and command of English were significantly related to using IT.

Given that it is inevitable to know English to interact with computers and every user who starts to work with computers will be familiar with numerous idioms, knowledge of English and skills will influence on computer litracy and will make it easy for users to work with computers.(25) Additionally, the results of Rahimi's study showed a positive meaningful relationship between the skills of English reading and internet searching. In their researh, individuals who were more proficient in English reading, used more the behavioral strategies, such as ensuring the process of searching, correct routing, and using advanced searches. They also used metacognitive strategies, like continuous assessment of information, selecting the main tiltles on webpages, following hyperlinks, and making decisions about saving and using more information.(26)

Our participants noted that the main facilitators in using the internet, in descending order, were ease and comfort in using computers and the internet, access to experts in case of any problems or questions, having internet courses as part of the curriculum, introduction to useful websites by professors or friends, and knowledge in using computers. Saeidi *et al.,*(11) listed the facilitators, in descending order, as feeling peace and ease of using computers, interest in using computers and the internet, computer and internet skills, good command of English, and access to experts in computer and internet fields in case of problems.

As for the deterrent factors in using the internet, distractions and lack of concentration while using the internet, internet connection problems (low speed, frequent disconnection, etc.,), internet costs, more reliability of the information provided by professors, books, and journals were mentioned in descending order by the participants. Jadoon *et al.,*(15) indicated that lack of time, inadequate number of available computers, and lack of support from the staff were the most common problems faced by students while accessing the internet in the institution facilities. In the study by Jafari and Diani,(4) the most important barriers for internet use were mentioned as: no internet access, being unfamilar with searching skills, lack of personal computers, high expense, failure of telecommunication lines, low-speed internet, and lack of need. Saeedi *et al.,*(11) mentioned low speed of internet connection, lack of access to a suitable computer, poor knowledge of information databases, lack of information searching skills, and poor English knowledge as the main problems of using the internet. Rihlert *et al.,*(27) indicated some other factors, like unfamiliarity with websites and lack of knowledge about the use of databases, as the most important barriers of internet use. Given the above, one may argue that having proper infrastructure to guarantee reliable access to the internet, decreased costs of internet connection, and holding training classes can motivate students to use the internet to find the latest scientific materials. 

## Conclusion.

In summary, it appears that learning how to use computers and the internet can be through self-practice and self-study, while professional use needs passing training courses. Therefore, there is a need for holding routine training courses in academic and scientific manner by experienced instructors. In addition, some courses can be designed as self-study and online education as a way to motivate students to use computers and the internet more often. Providing access to high-speed internet, regular training, and having updated information about computers, e-data sources, and information banks also promotes using the internet among students. Having access to experts in the fields of internet search can also be helpful for those facing problems with working with the internet. Given the key role of English knowledge, English courses should be followed more seriously in nursing faculties by designing more efficient courses. Future works should be conducted as experimental studies to examine the role of deterrents and facilitators in using computers and the internet.

## References

[1] Gündüz HB (2010). Digital divide in Turkish primary schools: Sakarya sample. TOJET.

[2] Mokhtarinoori J, Zohari S, Yaghmai F, Ebadi A, Yoldashkhan M. (2011). Study of factors relation to internet use with usage of internet by teachers according to theory of reasoned action. Iran J. Nurs. Midwifery Res..

[3] Jacobs HL. (2008). Information literacy and reflective pedagogical praxis.. J. Acad. Librarian..

[4] Jafari (2010). Evaluation of the use of the Internet by students at Kabul University and determinants of Facilitators and Barriers factors.. J. Libr. Inf. Sci..

[5] Deltsidou A, Gesouli- Voltyraki E, Mastrogiannis D, Noula M (2010). Undergraduate nursing students computer skills assessment: a study in greece. Health Sci. J.

[6] Hallila LE, Al Zubaidi R, Al Ghamdi N, Alexander G (2014). Nursing students' use of Internet and Computer for Their Education in the College of Nursing. Int. J. Nurs. Clin. Pract..

[7] Jetté S, Tribble DS-C, Gagnon J, Mathieu L (2010). Nursing students' perceptions of their resources toward the development of competencies in nursing informatics.. Nurse Educ. Today..

[8] Gilmour JA, Huntington A, Broadbent R, Strong A, Hawkins M. (2012). Nurses’ use of online health information in medical wards.. J. Adv. Nurs..

[9] Gilmour JA, Scott SD, Huntington N (2008). Nurses and Internet health information: a questionnaire survey. JAN.

[10] Rastgoo A, Naderi E, Shriatmadari A, Seifnaraghi M. (2011). The impact of Internet information litracy training on university student’s problem solving skills. Q. J. N. Approach Educ. Ad..

[11] Saeidi M, Yaghmaei F, Ranjbaran M, Godarzvand L, Hariri G, Imanzad M (2014). Knowledge, skills, access and usage status of computers, internet and databases in nursing students and some of the related factors. Adv. Nurs. Midwifery.

[12] Kumar S, Tadakamadla J, Tibdewal H, Duraiswamy P, Kulkarni S. (2010). Internet usage among undergraduate dental students in India.. Rev. Odonto Cienc..

[13] Abtahi S, Feali M (2008). Evaluation of skill and use of Information Technology and Internet among professors, postgraduate and undergraduate students in Mashhad Dental School in Iran in 2007-2008. J. Mash Dent. Sch..

[14] Jamshidi L, Mehrdad AG, Jamshidi S (2012). Assessing nursing students’ knowledge and attitudes about computers and the internet. Procedia Soc. Behav. Sci..

[15] Jadoon NA, Zahid MF, Mansoorulhaq H, Ullah S, Jadoon BA, Raza A (2011). Evaluation of internet access and utilization by medical students in Lahore, Pakistan.. BMC Med. Inform. Decis. Mak..

[16] Almarabeh T, Rajab L, Majdalawi YK. (2016). Awareness and usage of computer and internet among medical faculties’ students at the University of Jordan. J. Software Eng. Appl..

[17] Maleki Z, Goudarzi M, Mohtashami L, Faghihi B (2010). Knowledge and attitudes of dental students and academic staffs towards internet usage in dental training in Shahid Beheshti University of Medical Sciences. Shahid Beheshti Univ. Dent. J..

[18] Houshyari AB, Bahadorani M, Tootoonchi M, Gardiner J, Peña RA, Adibi P (2012). Information and communication technology in medical education: an experience from a developing country.. J. Pak. Med. Assoc.

[19] Unnikrishnan B, Kulshrestha V, Saraf A, Agrahari A, Prakash S, Samantaray L (2008). Pattern of computer and internet use among medical students in Coastal South India.. South East Asia Reg. Assoc. Med. Educ..

[20] Maroof KA, Parashar P, Bansal R (2012). How are our medical students using the computer and internet? A study from a medical college of north India.. Niger. Med. J..

[21] Mashhadi M, Rezvanfar A. (2007). Effective factors on IT application by agricultural and natural resources faculty members at Tehran University.. Q. J. Res. Plan. High. Educ..

[22] Sajadi FS, Shokoohi M, Kakoei S, Sheikhi F. (2013). Evaluation of skills and use of computer and internet among professors, postgraduate and undergraduate students in Kerman, Iran. Strides Dev. Med. Educ.

[23] Shirazi F, Sharif F, Molazem Z, Alborzi M (2017). Dynamics of self-directed learning in M.Sc. nursing students: A qualitative research. J. Adv. Med. Educ. Prof..

[24] Asadi A, Karimi A, Karami F. (2010). Identifying areas of information technology use by educators in teaching science.. J. Agric. Econ. Dev..

[25] Rahimi M, Yadollahi S (2011). Study on the relationship between highschool students’ computer anxiety with their field of study, gender, and English language achievement.. Q. J. Educ. Innov..

[26] Rahimi M, Hosseini M. (2013). Assesment relationship of information search with English reading skill.. Inform. Commun. Technol. Educ. Sci..

[27] Madsen-Rihlert C, Nilsson K, Stomber MW. (2012). Information Retrieval-Swedish Specialist Student NursesStrategies for Finding Clinical Evidence.. Open Nurs. J..

